# Rebound and Spillovers: Prosumers in Transition

**DOI:** 10.3389/fpsyg.2021.636109

**Published:** 2021-04-15

**Authors:** Elisabeth Dütschke, Ray Galvin, Iska Brunzema

**Affiliations:** ^1^Fraunhofer Institute for Systems and Innovation Research ISI, Karlsruhe, Germany; ^2^Institute for Future Energy Consumer Needs and Behavior, Aachen, Germany

**Keywords:** prosuming, rebound, spillover, psychological and economic drivers, socio-technical context

## Abstract

Generating energy by renewable sources like wind, sun or water has led to the emergence of “clean” energy that is generally available at low cost to the environment and is generated from seemingly unbounded resources. Many countries have implemented schemes to support the diffusion of renewable energies. The diffusion of micro-generation technologies like roof-top photovoltaics is one of the success stories within the energy transition and has been significantly driven—at least in countries such as Germany—by households. As these households usually not only generate energy but also consume it they are often called “prosumers.” How does it influence the energy behavior of households if they become prosumers? Are these behavioral changes in line with further goals of the energy transition, e.g., reducing demand? What shapes individual behaviors of prosumers? The paper introduces a conceptual framework based on the existing literature on rebound and spillover effects. It systematizes possible behavioral consequences as well as mechanisms behind them. This framework is then used to code and analyze data from 48 in-depth interviews with prosumer households. These interviews reveal a broad variety of behavioral responses which have their roots in economic conditions and their evaluation by the prosumers, psychological mechanisms like central guiding principles and a clear conscience as well as sociotechnical context and legislative frameworks.

## Highlights

- Private energy prosumers are a relevant group of active agents in the energy system
- To support the energy transition their behavior needs to align with demand reduction goals
- This interview-based study explores self-reported behaviors and how it emerges
- Behavioral response is heterogeneous and driven by individual and systemic factors.

## Introduction

The transformation of conventional energy systems that heavily rely on fossil fuels is a crucial element in strategies to solve humanity's current major challenge of achieving climate change mitigation goals and enhancing sustainability in order to stay within the limits of planetary boundaries. Increasing the shares of renewable energy, i.e., energy that is gained from resources like wind, water, and sun is one of the main pathways in the energy system transformation. Most prominent so far is the transition of the electricity sector by installing windfarms, biomass power plants, hydroelectric power stations, and photovoltaic (PV) panels. However, in addition to such a supply side oriented approach, all prominent scenarios for the transformation of the energy system also encompass the reduction of the demand for current energy services by increasing energy efficiency (e.g., IEA and IRENA, 2017). A well-known example for such a combined strategy is the 20-20-20-goals of the European Union (EU) which foresaw a 20% cut in greenhouse gas emissions (from 1990 levels), 20% of EU energy from renewables, and a 20% improvement in energy efficiency by 2020; the EU goals for 2030 were again defined in a similar way. Citizens and their investment decisions as well as their daily behaviors play an important role for the success of these scenarios. Micro-generation technologies have become available at decreasing prices and have found considerable support from policies like Feed-In-Tariffs (FITs) which made them a safe and profitable investment for many. Consequently, private investors including households are playing a significant role in this field and these households have become so-called “prosumers” who generate and use their own electricity in addition to feeding it into the grid. Thus, the role of households in the energy system has been enlarged and at the same time is subject to expectations with regard to system contributions, i.e., keeping their demand stable or reducing it while contributing to supply.

The main topic of this paper is to take a closer look at the interplay between households' understanding of their role in the energy system and their experiences and perceptions. Therefore, this paper takes a close look at prosuming households, i.e., households owning a photovoltaic (PV) system and their energy lifestyle. More specifically, we analyze how being a prosumer influences households' energy-related behaviors. As a frame of reference to address this question we draw on current streams of literature that analyze rebound and spillover effects. While mainstream *rebound effects* literature describes unexpected shortfalls in reductions in energy demand following an increase in energy efficiency of an energy service (Chitnis et al., 2014), the literature on *spillover* refers to broader behavioral changes when an environmental behavior triggers further changes in other behaviors (Nash et al., 2017). Thus, the two concepts describe two sides of the same coin as they both account for how prior behavior—in our case becoming a prosumer—influences later behaviors. From a normative perspective, spillover refers to the positive side of further increases in environmental behaviors, while rebound captures the downside of more demand and resource-use. In a first step, this paper investigates behavioral consequences using the rebound-spillover dimension as a normative anchor. Furthermore, as outlined in more detail below, both literatures have identified possible mechanisms underlying such behavioral consequences. Traditionally, economic approaches emphasizing changes in prices and available income have featured prominently in the literature (Dimitropoulos et al., 2018), but further researchers have also emphasized psychological mechanisms (Peters and Dütschke, 2016; Dütschke et al., 2018; Seebauer, 2018) and socio-technical configurations (Galvin, 2020).

A body of literature that investigates behavioral consequences of using renewable energy sources or more specifically installing PV systems has recently begun to emerge (Wittenberg and Matthies, 2016; Oberst et al., 2019; Qiu et al., 2019; Li et al., 2020). To build on this new stream this paper firstly advances a conceptual framework within which prosumer energy behavior can be evaluated. Secondly, it applies this empirically by drawing on 48 in-depth interviews with prosumer households in Germany. The interview data is analyzed with respect to (i) behavioral consequences of being a prosumer and (ii) underlying mechanisms to these behaviors.

The next sections first further develop the conceptual background by defining relevant terms and describing possible outcomes of being a prosumer. This includes a categorization of possible underlying mechanisms. We then present the empirical data, describing the methods for data collection and analysis before presenting findings. In the concluding discussion we refer back to the broader embeddedness of prosumers in the energy system as a system under transition.

## Conceptual Approach and State of Research

### Behavioral Consequences of PV Use

Energy behavior refers to broad categories, ranging from everyday routines which are usually mainly shaped by habits, social practices, learned schemata and situational cues and performed without much cognitive effort (e.g., turning on the lights) to conscious decision making processes of much lower frequency that involve more extensive evaluation of potential risks, benefits, and probable outcomes (e.g., buying a home, installing a PV). In comparison to the habitual daily behaviors such investment behavior is sometimes called one-shot behavior. Potential behavioral consequences of PV use refer to all these different types of behavior. With regard to the energy transition, all these behavior types could be beneficial in the sense that, for example, they could contribute to reducing energy consumption or the level of demand management by synchronizing supply with demand, or have adverse effects by increasing consumption.

#### Defining Rebound and Spillover

Research on rebound effects has traditionally mainly emerged from studying the effects of increases in energy efficiency. It refers to the phenomenon that often the implementation of an energy efficiency measure does not lead to the expected level of energy savings but these remain at lower levels (Sorrell, 2015). Quantifications of rebound effects are usually estimated by subtracting the ratio of actual savings to expected savings from one, or alternately expressed: they are the ratio between the shortfall in savings and the expected savings. Psychological approaches to the rebound effect agree with this definition in principle, but emphasize behavioral aspects and determinants (Dütschke et al., 2018). From their perspective the increase in energy efficiency is understood “as an intervention that interrupts previous routines and thereby leads to behavioral change in how the relevant product or service is used” (Dütschke et al., 2018, p. 5). If this behavioral change intensifies the use of an energy service, this is observed as a rebound effect. Often authors differentiate between direct and indirect rebounds depending on whether the increase in demand occurs in the same or another behavioral domain (Chitnis et al., 2014).

**Figure 1:**
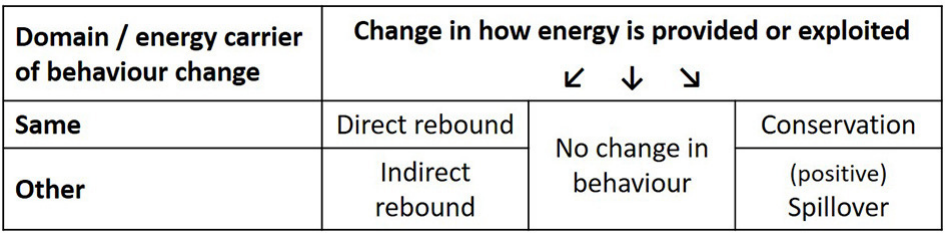
Overview of potential behavioral consequences of the way how energy is used or supplied.

May also occur in an opposite direction, and this is also supported by the literature reporting further reduction in demand or more broadly rising efforts of environmental behaviors (Truelove et al., 2014). The term (positive)^1^ spillover is used for effects in different domains (Galizzi and Whitmarsh, 2019), e.g., if the installation of a more efficient heating system is followed by electricity saving measures or triggers the purchase of a more efficient car. The rebound and spillover literatures have developed independently of each other, but have acknowledged each other's respective phenomena. For example, rebound literature has defined terms like reverse rebound (Chenavaz et al., 2021), prebound (Sunikka-Blank and Galvin, 2012), or super-conservation (Saunders, 2008; Li et al., 2020) to refer to situations where the actual energy demand falls below the expected. Similarly, research also refers to “permitting” or “negative” spillover to describe rebound-type effects (Galizzi and Whitmarsh, 2019). Taking the learnings from these literatures together, this paper combines the notion of rebound and spillover to describe the two sides of the same coin. To differentiate between effects in the same or other domains analogously to direct and indirect rebound effects, we will use the term conservation for effects in the same domain and spillovers for effects in other behavioral domains (see Figure 1 for an overview on the terms).

Transferring the definition of rebound effects to the field of renewable energy, a direct rebound effect in renewable energy use occurs if there is a higher demand for the same energy carrier when renewable energy is involved, compared to when no or less renewable energy is involved. In the case of household prosumers this would mean that the demand for electricity increases after installing a PV system, for example by buying additional appliances or using existing appliances more extensively. An indirect rebound effect of renewable energy use would occur if the demand for energy or other resources increases in other domains, e.g., an increase in travel or heating after installing a PV system.

In a similar vein, the concepts of spillover and conservation can also be transferred to the area of renewable energy use. The change to renewable energy would be said to trigger conservation if the demand is lower than before, e.g., if, after installing a PV, everyday usage behavior is changed such that lower electricity demand results (for example by turning lights off more frequently). Finally, there could be spillover to other domains, e.g., thinking about and actually implementing home insulation after installing a rooftop-PV.

Figure 1 takes up the notion that either increases in energy efficiency or a change to renewable energy supply could trigger behavioral responses, and summarizes the different effects.

#### Mechanisms Behind Rebound and Spillover

Economists have often associated rebound effect with price effects, i.e., if the usage of a service gets cheaper due to lower energy demand, then the demand for this service will increase (Dimitropoulos et al., 2018). These approaches usually do not consider the upfront investment but focus on the costs for obtaining the energy service. Applying this to the case of electricity generation with rooftop PV without considering the initial investment, the economics for the lifetime of the PV system strongly depend on the policy framework. In Germany, where our empirical case studies are situated, payments for renewable energy are governed by the Renewable Energy Law [Erneuerbare-Energien-Gesetz (EEG)]; the EEG has been revised several times and now incentivizes households to use the electricity from their PV themselves as this is cheaper than buying it from the grid. This can be maximized by households if they shift their consumption to times of (higher) generation. Thus, similar to the case of efficiency rebounds, prosumer households pay less for electricity services compared to non-prosumer households.

In addition to economic influences on behavior, the literature also suggests that psychological factors can foster or limit the emergence of rebound effects. This has to do with the degree to which needs are already satisfied (Hofstetter et al., 2006; Wörsdorfer, 2010), and norms and attitudes toward the relevant behavior and toward the environment (Haan et al., 2007; Matiaske et al., 2012). Peters and Dütschke (2016) proposed and empirically explored a conceptual model covering these concepts. Recently, moral licensing and consistency as explanatory factors have emerged in the literature (Dütschke et al., 2018). The moral licensing concept assumes that past morally positive behavior increases the probability that people will subsequently show potentially less moral behavior (Mazar and Zhong, 2010; Mullen and Monin, 2016). For behavioral spillovers, social and environmental identity have also been investigated (Elf et al., 2018; van der Werff and Steg, 2018; Verfuerth et al., 2019). Overall empirical research on these types of factors is rare so far, even more so in respect of renewable energy. From a conceptual point of view, all of the concepts under discussion seem highly applicable to also trigger rebound or spillovers in the case of renewables or more specifically the installation of a PV system. For example, studies have shown that investments in PV are likely to be regarded as environmental behaviors (Palm and Tengvard, 2011; Korcaj et al., 2015). These investments could thus provide a basis for a moral license, i.e., less environmentally friendly behavior and therefore lead to higher consumption. Alternatively, they could trigger consistent behavior, i.e., curtailment of consumption, by making an environmental identity or energy-related topics more salient.

While psychological approaches put a strong emphasis on individual control, they partly neglect the socio-cultural habitual embedding of behavior (e.g., learned behavioral patterns) (Galvin and Gubernat, 2016; Sonnberger and Gross, 2018) as well as socio-structural factors. Galvin (2013) elaborates on this for the example of windows: Most German windows are very badly designed for efficient manual ventilation by opening inwards in combination with the cultural habit of decorating window sills—thus this limits behavior that is ideal for energy efficient ventilation. In case of solar PV, the influence of such socio-technical structures is highly relevant in relation to the synchronization of supply and demand. Technical devices and ICT can support the synchronization which is otherwise limited to everyday heuristics by weather observations. However, such supporting technology is also likely to bring very specific conditions regarding practicalities that encourage or impede certain behaviors (cf. for example Wittenberg and Matthies, 2016 on the visibility in everyday life). The current German regulation on peak load prevention is an example of such a configuration for the case under study: it sets an incentive to use this electricity that prosumers otherwise perceive to be wasted. This could trigger households to make investments in, for example, electric mobility (bikes, cars) to make use of this electricity, and this could lead to higher demand overall (Galvin, 2020).

Other sources of rebound include lack of knowledge and technical or design failures. For example if PV modules are not set at an optimal angle, system components not optimally combined or settings of control units are wrong, this could lead to other energy demand patterns than anticipated. This can be due to lack of knowledge by users or installers, as well as the complexity of systems. A qualitative study by Peters and Dütschke (2016) found some evidence in this direction with regard to heating systems but also for lighting.

**Figure 2:**
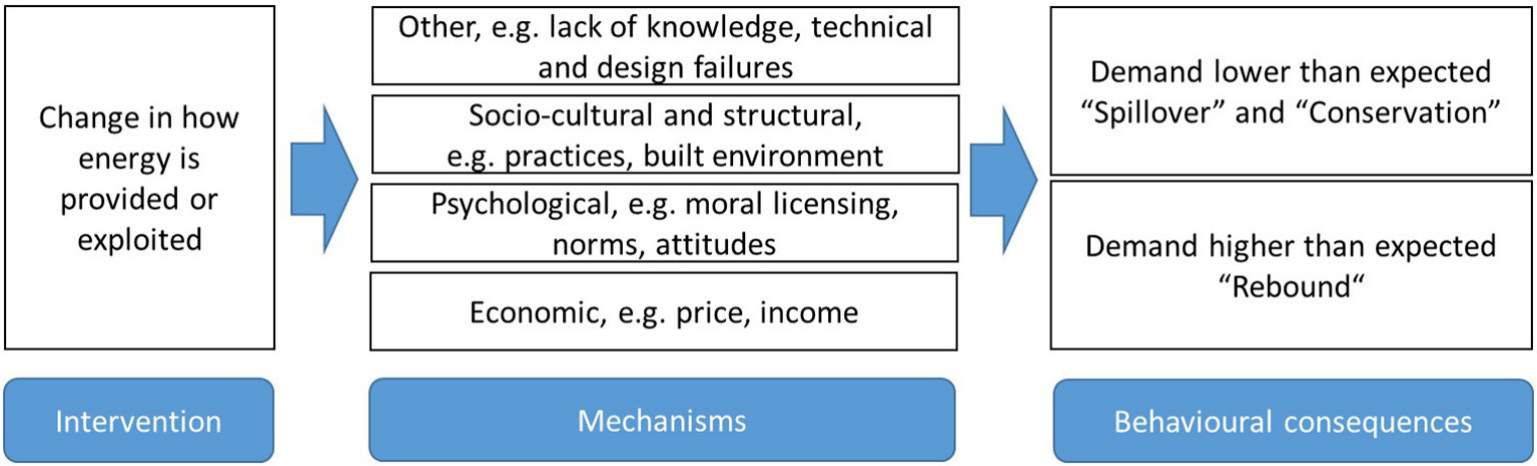
Schematic overview of potential behavioral effects and underlying mechanisms of efficiency increases. Own figure further developed from Dütschke et al. (2018).

Figure 2 summarizes the list of mechanisms identified from the literature.

### Empirical Findings in the Literature

The body of literature that examines potential consequences of small-scale PV on individual energy demand or more broadly of renewable energy use has only recently been emerging (cf. Luthander et al., 2015 for a review on earlier literature). The findings published so far cover a variety of samples studied by qualitative and quantitative approaches in different contexts and, thus, heterogeneous political and contextual factors. Consequently, the results vary substantially: Studies by Wittenberg and colleagues (Wittenberg and Matthies, 2016, 2018) used a German sample of more than 400 PV owners recruited by spreading the questionnaire through dedicated webpages. They obtained self-reported meter readings as well as questionnaire data. However, the quantitative analyses were limited in places due to missing data and small size of subgroups. Overall these two studies do not detect significant differences in consumption compared to general consumption in the population as reported in official statistics, but reveal support for a relationship between self-reported energy saving behaviors and positive environmental attitudes. Palm et al. (2018) interviewed 44 prosumer households in Sweden. These were recruited through a variety of sources, e.g., contacts from the energy agency, solar installers and advertisement on a blog. Participants were interviewed twice and reported their consumption data based on their entries in the web user interfaces of their electricity retailers. The researchers observed no major changes in consumption and hardly any indications of shifting demand according to electricity generation, but increasing energy awareness.

Qiu et al. (2019) obtained data from a utility company in the US including electricity meter data and survey data. In contrast to the studies cited before they estimated a solar rebound as high as 18 % by comparing the energy consumption of prosumer households with non-prosumers, and of 15% by comparing pre- and post-installation consumption (Qiu et al., 2019). The study also found effects of moderating variables, i.e., consumers from a neighborhood with more green/left wing voters showed smaller rebound effects. In a recent study, Li et al. (2020) who also combine metering and survey data find a small conservation effect for US PV prosumers who are financially incentivized to feed as much of their self-produced electricity into the grid as possible. Finally, Oberst et al. (2019) investigate energy use more broadly by analyzing self-reported heating costs for PV prosumer households and find no differences to non-prosumers.

Thus, the overall literature gives little or no consistent indication as to what (quantitative) extent the issue of rebound or spillover effects is relevant to PV households. The few studies available point out that there is variety among prosumers, and this appears to lead back to the categories of factors as identified in Figure 2. To enhance the state of knowledge we therefore explore the topic further through an analysis of 48 interviews with German prosumer households.

## Data and Methods

### Contextual Background

The study presented here is situated in Germany. PV panels are the dominant technology in this country for private self-generation of electricity. In 2016, around 8% of residential buildings in Germany were already equipped with a PV system, with the proportion particularly high for newer buildings, detached houses and buildings in southern Germany (Cischinsky and Diefenbach, 2018). PV generation is overall financially attractive for households, with high investments initially but very low running costs (Haar, 2020).

In 2018, around 20% of the renewable electricity generated in Germany was produced by PV, including large PV field arrays, and this contributed 7.7% to gross electricity consumption (ZSW and UBA, 2019). Of the installed capacity of German PV systems 15% falls into the category of up to 10 kWp and 34% in the range from 10 to 100 kWp (Wirth, 2020). After years of strong growth between 2005 and 2012 growth rates have slowed down (ZSW and UBA, 2019) since the policy and regulatory framework has changed.

German legislation mandates that the level of FIT at the time of installation of PV applies for 20 years. While the FIT for small-scale PV was around 57 ct/kWh for PV installed in 2004 it has constantly decreased since then and was around 11 ct/kWh for units installed in 2019 (Kelm et al., 2019). It is financially more attractive to feed PV electricity into the grid than to consume one's own electricity for households who installed PV up to 2012. For households who have installed PV since then, self-consumption is financially more attractive as the guaranteed FIT per kWh from then on became lower than the (average) price households pay for using electricity from the grid. This difference has constantly increased since then (Wirth, 2020). Thus, depending on when the PV system was installed, it is either more attractive for households to feed all electricity to the grid or more profitable to use it themselves, though with some differences regarding the precise economic benefit.

Under current legislation that was valid for the most recent interviewees as well as at the time of writing this paper, consuming self-generated PV electricity is free from electricity taxes and levies for PV installations below 10 kWp;^2^ but at higher capacities 40% of the regular EEG levy of a few cents must be paid per kWh consumed (EEG, 2017)^3^. However, the financial benefit from self-consumed electricity is subject to income taxes. Additionally, the current legislation guarantees a FIT of around 10 ct/kWh to households for the electricity they still feed to the grid if they do not use it themselves. In any case, consuming self-generated electricity is still cheaper than obtaining electricity from the grid, where prices are around 30 ct/kWh (BMWi, 2020). Thus, consuming electricity from a PV system installed by a household after 2012 leads to lower costs. Furthermore, to prevent grid overloads at peak generating times (e.g., midday in summer), PV system owners in Germany are obliged to allow grid operators to regulate their system (receiving lump-sum compensation for revenue lost); alternatively, smaller systems below 30 kW can limit their feed-in to 70% of their maximum effective power (EEG, 2017). Thus, for most households with recent PV installations, there is a limit to the amount that households can feed to the grid^4^.

### Description of Database

Four series of interviews serve as the database for this study. The total of 48 interviews were conducted in Germany between July 2017 and March 2019. They were obtained in four regional clusters and through a variety of recruitment procedures:

1. State of Hesse: The first series of interviewees was conducted between July and September 2017. The homes of the 13 respondents were mainly situated around the city of Darmstadt in the southern part of the State of Hesse which is at the center of Germany. Interviewers contacted potential participants by ringing at the door if PV systems were visible from outside or via internet maps. An earlier paper based on these interviews investigated the motivation to adopt a PV system (Köhler et al., 2019).
2. Wüstenrot: This small cluster was recruited at a citizen assembly and focused on inhabitants of an innovative building site at the small town of Wüstenrot, which is located in a rural area between the agglomerations of the cities Stuttgart and Heilbronn. In order to stand out and become attractive for potential citizens, the municipality has been pursuing local energy projects for some time. All houses on this newly developed housing estate were obliged to be equipped with a PV system. The homes are heated by an innovative heat network based on near-surface geothermal energy. Four households participated in the interviews which were conducted in March 2018. These were recruited at a citizen assembly for inhabitants of the housing estate.
3. Lower Franconia: 16 interviews with prosumer households in rural villages and towns around Schweinfurt in Lower Franconia, in the northern part of Bavaria, were conducted in February and March 2019. Interviewees were recruited through municipal newsletters, through a staff member of Schweinfurt County's energy support team and finally through local contacts of the authors. A paper focusing on other questions than those of the current paper is published by Galvin (2020) employing these interviews as a data source.
4. Markgräflerland around Freiburg: Finally another 15 participating households were recruited in the rural area around Freiburg in the southwest of Germany near the borders to France and Switzerland. Again, municipal newsletters were used to find interviewees, this was complemented by pre-identifying relevant homes through internet maps and ringing doorbells. The interviews were held in March 2019.

The interviews in cluster 1 were part of a psychology student research project and conducted under close supervision of the corresponding author. In Clusters 2 and 4 interviews were conducted by experienced interviewers from the corresponding author's institution. In cluster 3 the second author, who is also an experienced interviewer, social scientist and former electrical engineer, did the interviews. Originally, series 3 and 4 also included a few additional households who owned solar-thermal panels to heat water but no PV; they were excluded for reasons of consistency in the current paper.

The motivation behind combining these different clusters was to acquire a broad sample which is heterogeneous, for example with regard to local history and context including local discourses on renewable energy. This rationale was fueled by the aim that a qualitative study is appropriate when the goal is to further develop theory and enhance the in-depth knowledge on a topic. Thus, the main goal for sample composition is to make sure the full variety of the subject under study is captured. For this reason we also combined a variety of recruitment strategies, e.g., trying to acquire both more and less eager participants. The specific recruitment strategies were outlined above. Due to their heterogeneity it is not possible to estimate response rates.

The interviews were on the household level, i.e., in some cases more than one household member participated. More specifically the 48 households were represented by 32 men, three women, eleven couples and two women with their adult sons living in the same home. The average age of interviewees was 56, ranging from 27 to 82. Average household size was three with a range from one to seven. One third of the homes were situated in a town or city, two thirds in a rural area. We asked for self-ratings regarding income: one household saw themselves as below average, 20 as average, and 27 as above average. The solar panels were installed between 1999 and 2018 and thus cover the full range of the various FITs in this period. Nineteen households solely feed their electricity into the grid while a majority of 28 combine feeding into the grid with self-consumption and one household was not sure about this. For a detailed overview of the interview partners, see Appendix 2.

### Interview Topics and Analyses

The interviews were semi-structured, based on an interview guideline which was highly similar for clusters 1 and 2 and for clusters 3 and 4. The main difference in the interview contents of clusters 1/2 vs. 3/4 is that in the first two clusters a larger part of the interviews focused more extensively on the adoption process and how the decision for the PV system evolved; these interviews were on average also longer than in the second two clusters. The interview guideline for the second clusters is given in the Appendix to this paper. Besides the adoption process, the guideline featured details about the PV system and technologies, investments and systems for monitoring connected with it; the motivation and aims for the installation and discussions in the household around it; energy behavior before and after installing the PV; and questions about the local context. All interviews were accompanied by a short written questionnaire to assemble some key data about the household and its composition, electricity consumption, and PV system. The interview conversations were digitally recorded and transcribed verbatim. The text corpus of these transcripts adds up to an amount of 280,310 words overall.

As indicated above, parts of the interviews have been analyzed for different research questions. For the research interests under study in the present paper a coding frame was developed including main codes and subcodes (see Table 1) by applying content analysis starting with a theory driven deductive approach and refining the coding scheme inductively where necessary (Mayring, 2015). First, the interviews from series 1 and 2 were coded by the first author using a simplified coding scheme on behavioral consequences, extracting (1) further (intended) investments, (2) behavioral changes regarding electricity use, (3) synchronicity of consumption behavior with the sun, and (4) behavioral changes in other domains like water, transport, In this first analysis the lines of arguments by which households explained their respective behaviors and the behavioral outcomes were not separated from each other but subsumed using the same main codes. In a next step, the third author of the paper coded all interviews from all four series with a focus on the behavioral consequences. The main codes in this step were the same as above excluding quotes on underlying mechanisms and extending the behavior change category also to explicit statements that behaviors have not changed. In this step the code assignment in the first cluster was also checked for diverging interpretations, and high levels of agreement emerged. Finally, the first author refined the coding on behavioral consequences by going through all interviews again and additionally coding the underlying mechanisms. In a next step, the quotes on the subcodes were extracted by the first author and densified according to themes to allow for counting frequencies where applicable, e.g., regarding the technologies the households invested in.

**Table 1:** Overview on main codes and sub-codes applied to the interview data.

**Main code**	**Sub-code**
**Behavioral consequences**	
Energy system investments	Realized further investments Future options Denied investments
Daily behaviors: Synchronizing electricity demand with supply	
Daily behaviors: Behavioral change in electricity consumption	Reduction of demand Increased demand No change More conscious consumption
Daily behaviors: Behavioral change in other domains	Reduction of demand Increased demand No change More conscious consumption
**Mechanisms behind behavioral change**	
Individual level mechanisms	Economic, psychological
Socio-technical mechanisms	
Other	

The main codes are based on the concepts included in Figure 1 and displayed in Table 1. The coding process and the interpretation of results was also checked by the second author who was the interviewer of the (relatively large) Franconia study for consistency and plausibility. For a fuller account of issues that arise in coding to a high degree of reliability together with reviews of recent literature on this see O'Connor and Joffe (2020).

In the following, where quotes are provided from the interviews they are given by letters symbolizing the region, i.e., HE, Hesse; WÜ, Wüstenrot; FRAN, Franconia; FR, Freiburg, and a number identifying the relevant interview in the sample.

## Results

### Behavioral Consequences

Our analysis on behavioral consequences will start by outlining the findings on energy system investments. In this category we summarize investments *in addition to the PV*, that households made to save energy, to make better use of the electricity from the PV or replace the use of less sustainable energy sources, e.g., buying an electric car instead of a conventional one. This will be followed by an analysis of daily behaviors starting with (i) issues around synchronizing demand with supply, (ii) electricity use more generally and finally (iii) behaviors around energy and resource use more broadly.

#### Energy System Investments

In many respondent households the PV system is not the only step toward active integration into the energy production system or the uptake of relevant innovations. Overall, three quarters of the interviewed households (36 out of 48) have made additional investments in further technologies. On average this encompasses two further investments per household, ranging from 1 to 5. Most prominent is the use of a solar thermal system (15 households), battery storage (9), or a heat pump (8). Seven report that they implemented high insulation standards, including passive house standard in some cases; seven interviewees state that they use a sustainable heating system, e.g., running on wood or as a combined heat and power unit. Overall, ten use alternative drives for their vehicles, most prominently full electric cars (5). Further investments include highly efficient household appliances and lighting, water re-use systems, and smart home equipment. The timing of these investments and how they relate to owning the PV is often not fully clear in the interviews. Many interviewees describe them as different stations of a longer journey:

*HE4: As you said, it has always been important to us that we are aware of energy issues and we enjoyed having this possibility that we can contribute to exploiting the sun*.
I: Has that changed over time or increased?
HE4: Well, it expanded into other areas and we are now driving a hybrid car

Even more interviewees (34) elaborate on future investments. On average interviewees had two further ideas as to what such investments could be. Among these, adding battery storage (18) and/or buying an electric vehicle (15) are the dominant themes. For those feeding all their electricity to the grid, the next anticipated step is to move to self-consumption. Further ideas are similar to those already implemented and include sustainable heating systems, smart home elements, and micro wind turbines. The reasons these ideas have not yet been implemented are heterogeneous—in about a third of cases intentions are still vague and more in the stage of first ideas. That the necessary investment is considered as too high also plays a role. In some cases, the intention is firm but households are waiting for the right point in time, i.e., when the current car gets too old, the heating system breaks down or their guaranteed FIT is about to end.

Fourteen interviewees also excluded certain investments: five had turned down the option of buying electric cars due to restricted range, environmental reasons, or high prices; four were generally skeptical about battery storage, again due to high prices or an insufficient economic rationale as well as doubts that decentralized storage is beneficial to the energy system. Further ideas that were turned down by one of the households included more sustainable heating systems or home renovations, the main reason being too high initial investments.

#### Daily Behaviors: Synchronizing Electricity Demand With Supply

Many of the interviewees reported some degree of synchronizing electricity demand with sunshine. However, the majority of these are from the subgroup that is engaged in self-consumption. Of those fully feeding to the grid only two out of 19 households engage in synchronizing behaviors compared to 22 out of the 28 who do not fully feed into the grid. The main synchronizing activity is to aim at using basic household appliances like washing machines, driers, and dishwashers when the sun is shining or at least during daytime. Very few combine this with setting timers or some sort of home automation, i.e., these activities are mainly performed manually and the women in the households are often the ones implementing it, with the men often presenting themselves as the ones pushing in this direction:

I: So it is also in her blood that she [his wife] will turn on the washing machine or dishwasher in four hours or something like that?
*FR11: Yes, she does that. Because that's just a requirement of the boss [i.e. the interviewee]*.
I: Do you urge her or does that come from her?
*FR11: No, no. She already realizes that it makes sense. (…) [However,] if it doesn't fit and [she] just wants to have it done in the evening so that it is clean in the morning (…) then it must be possible to do that without the sun shining*.

The quote also points out limitations that are repeatedly mentioned, i.e., that synchronicity ends where it puts too much strain on comfort or interrupts necessary activities. This also refers to activities which interviewees do not consider shifting, such as cooking.

#### Daily Behaviors: Changes in Electricity Consumption

Codings around possible changes in daily behaviors regarding electricity consumption fall into four groups: (i) respondents reporting that they have reduced their electricity consumption due to or following the installation of the PV, (ii) households reporting increased consumption, (iii) statements indicating no change in consumption and finally, (iv) interviewees talking about an increase in awareness without stating or knowing the influence on actual consumption. These categories are not necessarily exclusive, i.e., the sample includes eleven people each making statements that fall into more than one group. For example, households explained about using more electricity in one case and less in another. Twenty one households only gave statements from just one of these categories. Hardly any of the interviewees were able to provide precise quantitative observations comparing the development before owning the PV and since then. Some had incidental data about yearly consumption, but, major changes were mainly due to children moving out. Some households started to constantly monitor their consumption since they own the PV. Twenty reported they used less electricity now and described themselves as frugal.

*HE12: And to always check where you can save more, or where you could use an energy saving lamp, or where you can replace a device with something that uses less energy. Of course always in a reasonable manner. You also need energy to produce the device, so to buy a new refrigerator for one kilowatt, that would be nonsense*.

However, many of these statements remained very general, sometimes alluding to turning off lights or reducing standby consumption.

Thirteen made statements describing perceptions that nothing has changed:

*FR13: I think nothing has changed. It is not that I now for example produce electricity and say, I can then waste all the more somewhere else. (I: Yes.) My behavior has not really changed because I now produce electricity myself and do not store anything. (I: Yes.) Nothing has changed. (I: Exactly.) Definitely not*.

In some cases further explanations about this lack of change go in different directions—either pointing out why reductions are not perceived to be necessary or, contrastingly, how the household just continued their always frugal lifestyle:

*FRA6: I say that you have a certain quality of life, and you don't really need to restrict it because the sun makes enough energy, yes*.
I: Yes. Do you think that over time, over the last 20 years, you have become more energy efficient, or about the same?
*FRA16: I think I have actually always been*.
R: Always?
*FRA16: Yes, I think strangely enough yes*.

Some (7) explain that the PV has increased their awareness:

*FR5: You just perceive it much more consciously. Because I get feedback on my energy consumption every day, I am much more aware of it. And I also realize what consumes energy at all and what doesn't*.

Finally, a smaller group (5) outline that their demand has increased. This is mainly bound to the acquisition of additional appliances and gadgets like garden lights, a fountain, or a solarium to get tanned. For some, the investment in PV was a response to high demand:

*FR3: So we were angry about our [electricity] bill. (…) We have a swimming pool inside and sand filter and that was close to 2000 Euro per year. And then we said: Well, that doesn't have to be. We wanted to reduce that*.

#### Daily Behaviors: Behavioral Change in Other Domains

Statements on further behavioral change in other domains than electricity were also given. One topic that repeatedly came up was travel behavior and more specifically flying. Several interviewees were very conscious about this topic and brought it up themselves. A small group made statements that they had given up flying a long time ago and do not intend to do so now, or explain about very specific exemptions from this principle (e.g., a couple working for the church flying to Israel for once in their life). Others claimed to make very conscious decisions regarding flying. However, there was also some variety as to what “flying rarely” means:

*FRA5-wife: Or, we also take a lot of vacations by bike. And often we go there by car. And if we deliberately go to vacation apartments, we have contact to the landlords. And, but we do take an airplane trip in winter (laughs)*.
FRA5-husband: Rarely. Every 2 years on average. But not a long distance trip, but sometimes to the Canaries or
FRA5-wife: Still little
*FRA5-husband: We want to go to Crete now. Or we went to Sicily now last year*.

In a similar vein, ambivalence about modes of travel extends to the choice of transport mode in daily life or the extent of car use.

Another area of resource use that is repeatedly mentioned is the use of water, with some households reporting about their installations for using rain-water or re-using e.g., water from showering for the toilet. Another topic is sparse or very conscious consumption when buying goods, reduced number of appliances, recycling, or reducing waste. Overall, interviewees give more examples of reduced or very conscious use of resources and fewer examples of high resource use levels. Of those who did speak of high resource use levels, two households reported heating over-generously.

### Mechanisms Behind Behavioral Change

The underlying mechanisms that interviewees refer to are broad and heterogeneous. For some the investment in the PV system is already described as one step that was logical from what they had thought and experienced earlier and which also led them on to further investments and/or consistency in their daily behaviors (cf. quote from HE4 above). As outlined before, the PV investment is sometimes followed by behavioral changes, sometimes the PV is installed in response to behavioral change or high demand (e.g., maintaining a swimming pool FRA4). This will be described in more details in the remainder of this section. When the coding scheme was developed it also included the category “other” (cf. Figure 1 and Table 1), however, this subcode did not turn out to become relevant.

#### Individual Level: Economic and Psychological Mechanisms

Economic vs. environmental motivations are the dominant areas of discussion (often contrasted by interviewees). Some state a clear dominance of the one or the other or emphasize both, in other cases motives and how they actually influence decision making and daily behaviors seem less clear. For some, saving money is an important mechanism that drives them to synchronize their consumption patterns with the sunshine.

In some cases, the economic outcome was not clear at the point of time of decision making, with high initial investments, and was evaluated positively when people realized that later.

However, some directly reject economic thinking:

FRA2: When you buy a Mercedes with leather seats, do you ask if the Mercedes with leather seats is profitable or if a Golf with cloth seats is profitable? Does anyone ask, if one builds a dormer, if this dormer is profitable or if a roof window would be sufficient?

In these cases, the decision on the investment for the PV and further technologies depended on the affordability, but not on anticipated financial gains.

Furthermore, the interviews indicate a variety of guiding motivations (“Leitmotif”) that some interviewees refer too, often repeatedly, during the interview and connecting different behaviors and decisions following this *Leitmotif*. One of them is autarchy, i.e., some interviewees explain their investment in PV and also additional investments like storage by their desire to become independent of the energy system, and also of changing prices.

*FRA7: I think there is a high vulnerability of our systems that we are not aware of today and the idea that I can get an emergency power supply from my own - my own energy storage and my photovoltaic system - is already a motivation to invest even more money*.
*WÜ1: I don't care how much the oil costs (…) I always have mine somewhere and as I said, I can influence it myself, just very well by simply orienting myself a little towards the sun, so that's a great thing*.

In some cases the themes of sustainability and/or environmentalism are playing an important role across different situations:

*HE7: So, as I said, I wanted to do something for the environment. And of course that's one aspect, decentralized energy generation. There are many other environmental things you can do (…). Not driving a car, for example, is one. [laughs] Well, I'm also a cyclist, just by the way*.

Some households are proud and enjoy what they achieved in this regard:

*HE12: So the feeling is that the electricity I consume here, it is also fun with such an attitude as mine to consume as little as possible*.

This goes as far that the enjoyment in everyday life is described in vivid pictures:

*FRA7: I have now already told my wife that it is a completely different feeling to shower with solar heat, with solar thermal water. (…) Not a lot of oil runs down over your head but solar heat runs over your head*.

Others that emphasize ecological motives focus on increasing awareness as an ongoing process as pointed out above.

Finally, in one case, frugality *per se* is described as the guiding principle.

A different psychological mechanism in addition to the leitmotif that emerges in several interviews is the idea of having a clear conscience due to using solar energy. In some cases this clear conscience is then used to justify behaviors that are not fully sustainable like traveling or using more energy/electricity.

#### Socio-Technical Mechanisms

The legislative framework also plays a role in shaping the behaviors of PV households. As pointed out earlier, in few cases further investments are currently held back as households still enjoy a high FIT and do not want to change the configuration before it ends. In one case, the household chose a smaller PV to stay beyond a certain limit in the regulations.

Regulatory and sociotechnical influences can sometimes be closely interwoven. One of the peculiarities of the German legislation on renewable energy is that to prevent peak loads, PV system owners in Germany are obliged to allow grid operators to regulate their system (receiving compensation for it); alternatively, smaller systems below 30 kW can limit their feed-in to 70% of their maximum effective power (EEG, 2017). Thus, there is a limit to the amount that households can feed to the grid^5^. This is only relevant to newer systems as this rule is relatively new. Those affected by it in our sample often refer to it and some are deeply concerned to find ways to use the relevant electricity and prevent it from being “wasted.”

Another topic at the interplay between technology and household behaviors is how the actual supply with electricity is monitored, if at all. Some “monitor” the system only scarcely by checking if the light of the control unit is still on when they pass by.

*FRA19: So technology is—I must say—I am from the humanities. I'm really not interested in technology. Not very, huh? (…) I look at my equipment working in my basement. I can see whether the green light is on or not (laughing)*.

Others are in the position to access real-time information about current supply and battery status (if applicable) through smartphones and similar devices and also report that they observe this closely and also use it to educate other household members. Others have established a paper-and-pencil monitoring, often on a monthly basis to detect larger deviations.

*HE3: For me, I do it in my book, in which I enter my consumption and production every month, just like with water and gas, and I have sensitized my children to the point that they are happy if they consume less themselves*.

## Discussion and Conclusions

This paper started out to take a closer look at how prosuming changes energy-related behavior of households. As a conceptual framework we drew on the literatures on rebound and spillover and as an empirical basis on 48 interviews with prosumers from four regional clusters. The focus of prosumers is motivated by the fact that prosuming households are an example how the energy transition as part of the great transformation toward sustainability manifests on the individual level. By generating electricity, households change their role in the electricity system and leave behind being passive consumers. It is against this background that we take a detailed look at how prosumers describe their interactions with the PV system, if, how and why it changes their energy behaviors.

What we found in the interviews is a broad variety of behavioral responses. Further investments, already realized or planned for the future, play a prominent role. Many households have already combined their PV with further technologies or have thought a lot about how to do so in the future. To some extent this resonates with the finding of Cohen et al. (2019) as there appears to be “q-complementarity” between investment in PV and in certain other electrical goods. Q-complementarity is said to occur when the welfare gain from adopting one good is increased by the welfare gain from adopting another good and vice versa. Becoming a prosumer is for many of our respondents not an end in itself but just one step in a longer journey. In this vein, the investment in the PV system not only impacts future investment decisions, but was often triggered by earlier experiences.

The behavioral responses in daily routines are also heterogeneous within the households. Some quite clear cases of consistent environmental concern and motivation throughout emerge, and others where environmental concern and environmentally supportive behavior were gradually amplified through their experience of having PV. Furthermore, for some the PV is a kind of compensatory investment as they perceive their consumption as exceptionally high. Finally, for some the PV is also a means to justify increases in demand or luxury investments, one of the impressive examples is probably the household that added a solarium.

The mechanisms that trigger the behavioral responses are also broad and heterogeneous, and economic, psychological and socio-technical drivers were sometimes closely interwoven. At the same time, drivers do not seem to unfold homogeneously or consistently. For example, economic mechanisms act as an important driver to some, but others highlight the relevance of affordability rather than economic viability). Psychological issues were mainly revealed in the form of guiding principles (leitmotif), and less as specific relationships between psychological variables like norms or attitudes. Having a “good conscience” was emphasized in some interviews and points to the relevance of moral issues, i.e., licensing or consistency behaviors.

The link to the energy transition shows up most via socio-structural mechanisms, and these relate most strongly in our analysis to the embeddedness of prosumers and their PV in the electricity system and its regulatory framework. We find different types of effects of the legislative context which give important signals to prosumers; however, the perceived influence of regulations seems sometimes higher than their actual relevance. For example, some of the households are concerned about the energy they are not allowed to feed to the grid due to a recent cut-off rule. However, technical estimations indicate that the actual loss is likely to be small (Wirth, 2020). Thus, it seems likely that many households cannot draw on exact economic or technical estimations (due to lack of knowledge, interest and/or data). Rather, the regulative structure provides rules of thumb which are then translated into behavioral heuristics.

Pointing to the limitations of the paper, it seems highly likely that different findings would emerge in different national contexts, e.g., where financial incentives and regulatory contexts differ. In our case this is mirrored by the differences between people fully feeding into the grid and those who consume some of electricity themselves. Another limitation is that due to the semi-structured guideline there is variation between the interviews as to which topics came to the fore and which did not. Thus, it is possible that some issues or mechanisms play a role for further households but did not enter the discussion during any of the interviews. This is especially likely to apply to behavioral consequences beyond electricity use where it might have been difficult for interviewers and interviewees to touch upon all possible topics.

This paper adds to the literature by giving a very detailed and thereby innovative account of the behavioral consequences of adopting PV and why these emerge. Some of the findings are in line with earlier literature that pointed to increases in awareness (Palm et al., 2018). Furthermore, the broad variety of behavioral responses also fits with the heterogeneity of past findings regarding the emergence and size of potential rebound or conservation/rebound effects (Oberst et al., 2019; Qiu et al., 2019; Li et al., 2020): Households brought forward a variety of logics and descriptions to explain their behaviors. This in-depth account of qualitative findings can inform the design of future quantitative studies that build on our findings. Large samples would also allow for subgroups, so that the full context could be better grasped and considered via rigorous statistical analysis.

## Data Availability Statement

The datasets presented in this article are not readily available because the interview data is impossible to be anonymized. Requests to access the datasets should be directed to Elisabeth Dütschke, elisabeth.duetschke@isi.fraunhofer.de.

## Ethics Statement

Ethical review and approval was not required for the study on human participants in accordance with the local legislation and institutional requirements. The patients/participants provided their written informed consent to participate in this study.

## Author Contributions

ED wrote a first draft of the paper and revised it based on co-authors comments and was strongly involved in the data analysis. RG conducted one of the interview series, commented extensively on earlier versions of the paper and edited the text. IB was involved in coding the data and provided comments on the paper. All authors contributed to the article and approved the submitted version.

## Conflict of Interest

The authors declare that the research was conducted in the absence of any commercial or financial relationships that could be construed as a potential conflict of interest.

## Footnotes

^1^Sometimes the literature differentiates between positive and negative spillover. Negative spillover effects are conceptually identical to the concept of indirect rebound effects (Nash et al., 2017).

^2^Although the marginal cost of producing each extra kWh is zero (Haar, 2020), there are still maintenance costs for PV and its electrical circuit technology. Most of these are usually very low, but storage batteries and DC-AC converters are expensive to replace if they fail.

^3^In 2020, this was 2,7 €ct / kWh, cf. Bundesnetzagentur (2020).

^4^As the real output is rarely higher due to weather and technical conditions, the actual resulting loss is only about 2-5%, cf. Wirth (2020).

^5^As the real output is rarely higher due to weather and technical conditions, the actual resulting loss is only about 2-5%, cf. Wirth (2020).
