# Supplementary material for: Rebound and Spillovers: Prosumers in Transition
Source: Front Psychol. 2021 Apr 15;12:636109. doi: 10.3389/fpsyg.2021.636109 (PMC8082245; doi:10.3389/fpsyg.2021.636109)
Supplement: Supplementary file 1 [file Data_Sheet_1.docx]

Appendix

# Appendix 1 - English translation of interview questioning

Note that this is a guide for leading a two-way discussion, not a simple question-answer session. All the questions should be asked, but the interviewer is to allow the interview-ee(s) to pursue these or other or related issues as they arise in their minds and to follow these up with deeper probing as appropriate. This also means the order of questions could be adapted to how the interview evolved.

Motivation and decision making

- What was your motivation for installing PV/solar thermal energy?
- How did the decision-making process go? (family meeting, trigger, etc.)
- What kind of companies or organizations were there, to provide information, and did you use them?
- What was important for the decision, what tipped the scales?
- How does it make you feel that you have PV/solar thermal on your house?

Practices (within and outside the home)

- Do you think that the fact that you have a PV-ST system influences your daily life at home? during energy consuming activities? in decision-making processes and dis-cussions at home?
- Is there a strategy for energy management in the household?
- And (the above regarding) your everyday life outside the home (mobility, holidays, work, leisure, etc.)?
- Has having PV/ST brought any changes to your life?

Money

- Financing: how did you finance the installation of the PV/solar thermal energy (loans, savings, etc.)
- Does this have an impact on how you consume energy or use household applianc-es?
- Does it affect other aspects of your life, inside or outside the home?
- Have you calculated the profitability of the installation? Was this a factor?

Environment, attitudes

- You often hear that having PV on the roof is very environmentally and climate friendly. What do you think about this?

Knowledge and awareness

- To what extent do you think you understand the technology of your PV/ST plant and its control system, etc.?
- To what extent do you think you understand the monetary aspects of your PV/ST system? (savings, compensation, tax, etc.).
- Does a person need to be a professional to use your PV/ST system economically and efficiently?
- Do you record your energy production, use or self-production use? If so, in what ways?

Regional and cultural issues

- Do you think it is particularly important for households in X to have PV / solar thermal energy?
- What role do households in X with PV / solar thermal energy play?

Technical aspects

- How would you describe your PV/ST system? (Store electricity? Self use? kWP? kWS/year?
- Does the technology associated with your system help or hinder efficient and effec-tive use of energy? In what ways?

# Appendix 2 - Interview Partners

| **ID** | **gender** | **age** | **household size** | **Self-estimated income category** | **urban/**  **rural** | **year of  installation** | **energy use** |
| --- | --- | --- | --- | --- | --- | --- | --- |
| HE01 | m | 73 | 2 | average | rural | 2002 | infeed |
| HE02 | m | 60 | 1 | above average | rural | 2008 | infeed |
| HE03 | m | 60 | 2 | average | urban | 2011 | self-consumption and infeed |
| HE04 | m/f | 55/na | 5 | above average | rural | 1999 | infeed |
| HE05 | m | 52 | 3 | average | rural | 2016 | self-consumption and infeed |
| HE06 | m | 51 | 5 | above average | rural | 2014 | self-consumption and infeed |
| HE07 | m/f | 61/na | 4 | above average | urban | 1999 | infeed |
| HE08 | m/f | 55/na | 4 | average | urban | 2016 | self-consumption and infeed |
| HE09 | m | 50 | 4 | above average | urban | 2014 | self-consumption and infeed |
| HE10 | m | 40 | 4 | average | urban | 2017 | self-consumption and infeed |
| HE11 | m | 66 | 2 | above average | urban | 2015 | self-consumption and infeed |
| HE12 | m/f | 57/na | 2 | average | urban | 2007 | infeed |
| HE13 | m | 55 | 3 | above average | rural | 2011 | self-consumption and infeed |
| WÜ01 | m | na | na | average | rural | 2013 | self-consumption and infeed |
| WÜ02 | m | na | na | above average | rural | 2014 | self-consumption and infeed |
| WÜ03 | m | na | na | average | rural | 2017 | self-consumption and infeed |
| WÜ04 | m | na | na | above average | rural | na | self-consumption and infeed |
| FRAN01 | m/f | 46/48 | 2 | average | rural | 2009 | infeed |
| FRAN02 | f | 53 | 3 | above average | rural | 2017 | self-consumption and infeed |
| FRAN03 | m | 62 | 2 | above average | rural | 2018 | self-consumption and infeed |
| FRAN04 | m | 52 | 5 | above average | rural | 2004 | infeed |
| FRAN05 | m/f | 73/62 | 2 | above average | rural | 2000 | infeed |
| FRAN06 | m | 63 | 1 | average | rural | 2013 | infeed |
| FRAN07 | m | 57 | 2 | above average | rural | 2001 | self-consumption and infeed |
| FRAN08 | m/f | 72/67 | 2 | average | rural | 2013 | self-consumption and infeed |
| FRAN09 | m/f | 35/33 | 5 | above average | rural | 2016 | self-consumption and infeed |
| FRAN10 | m | 60 | 3 | average | rural | 2008 | infeed |
| FRAN11 | m | 51 | 4 | average | rural | 2003 | self-consumption and infeed |
| FRAN12 | m | 63 | 4 | above average | rural | 2000 | infeed |
| FRAN13 | m/f | 27/67 | 2 | average | rural | 2001 | infeed |
| FRAN14 | m | 44 | 5 | above average | rural | 2016 | self-consumption and infeed |
| FRAN15 | f | 66 | 1 | average | rural | 2005 | infeed |
| FRAN16 | m | 52 | 5 | below average | rural | 2001 | self-consumption and infeed |
| FR01 | m | 74 | 2 | average | urban | 2008 | infeed |
| FR02 | m | 45 | 4 | above average | urban | 2014 | self-consumption and infeed |
| FR03 | m/f | 27/67 | 3 | above average | urban | 2008 | infeed |
| FR04 | f | 44 | 3 | above average | rural | na | na |
| FR05 | m | 41 | 5 | above average | urban | 2017 | self-consumption and infeed |
| FR06 | m | 47 | 5 | above average | na | 2009 | self-consumption and infeed |
| FR07 | m/f | 71/na | 2 | average | rural | 2012 | self-consumption and infeed |
| FR08 | m/f | 82/na | 7 | average | rural | 2012 | self-consumption and infeed |
| FR09 | m | 63 | 2 | above average | urban | 2008 | infeed |
| FR10 | m | 39 | 5 | above average | urban | 2011 | self-consumption and infeed |
| FR11 | m | 41 | 4 | above average | urban | 2015 | self-consumption and infeed |
| FR12 | m/f | 79/na | 2 | average | urban | 2009 | self-consumption and infeed |
| FR13 | m | 74 | 2 | above average | rural | 2001 | infeed |
| FR14 | m | 70 | 2 | average | urban | 2008 | infeed |
| FR15 | m | 50 | 4 | above average | rural | 2008 | infeed |
